# *In-Silico* Tool for Predicting, Scanning, and Designing Defensins

**DOI:** 10.3389/fimmu.2021.780610

**Published:** 2021-11-22

**Authors:** Dilraj Kaur, Sumeet Patiyal, Chakit Arora, Ritesh Singh, Gaurav Lodhi, Gajendra P. S. Raghava

**Affiliations:** ^1^ Department of Computational Biology, Indraprastha Institute of Information Technology, New Delhi, India; ^2^ Department of Computer Science, Indraprastha Institute of Information Technology, New Delhi, India

**Keywords:** innate immunity, defensins, AMPs, computer aided, machine learning

## Abstract

Defensins are host defense peptides present in nearly all living species, which play a crucial role in innate immunity. These peptides provide protection to the host, either by killing microbes directly or indirectly by activating the immune system. In the era of antibiotic resistance, there is a need to develop a fast and accurate method for predicting defensins. In this study, a systematic attempt has been made to develop models for predicting defensins from available information on defensins. We created a dataset of defensins and non-defensins called the main dataset that contains 1,036 defensins and 1,035 AMPs (antimicrobial peptides, or non-defensins) to understand the difference between defensins and AMPs. Our analysis indicates that certain residues like Cys, Arg, and Tyr are more abundant in defensins in comparison to AMPs. We developed machine learning technique-based models on the main dataset using a wide range of peptide features. Our SVM (support vector machine)-based model discriminates defensins and AMPs with MCC of 0.88 and AUC of 0.98 on the validation set of the main dataset. In addition, we created an alternate dataset that consists of 1,036 defensins and 1,054 non-defensins obtained from Swiss-Prot. Models were also developed on the alternate dataset to predict defensins. Our SVM-based model achieved maximum MCC of 0.96 with AUC of 0.99 on the validation set of the alternate dataset. All models were trained, tested, and validated using standard protocols. Finally, we developed a web-based service “DefPred” to predict defensins, scan defensins in proteins, and design the best defensins from their analogs. The stand-alone software and web server of DefPred are available at https://webs.iiitd.edu.in/raghava/defpred.

## 1 Introduction

**Figure 1:**
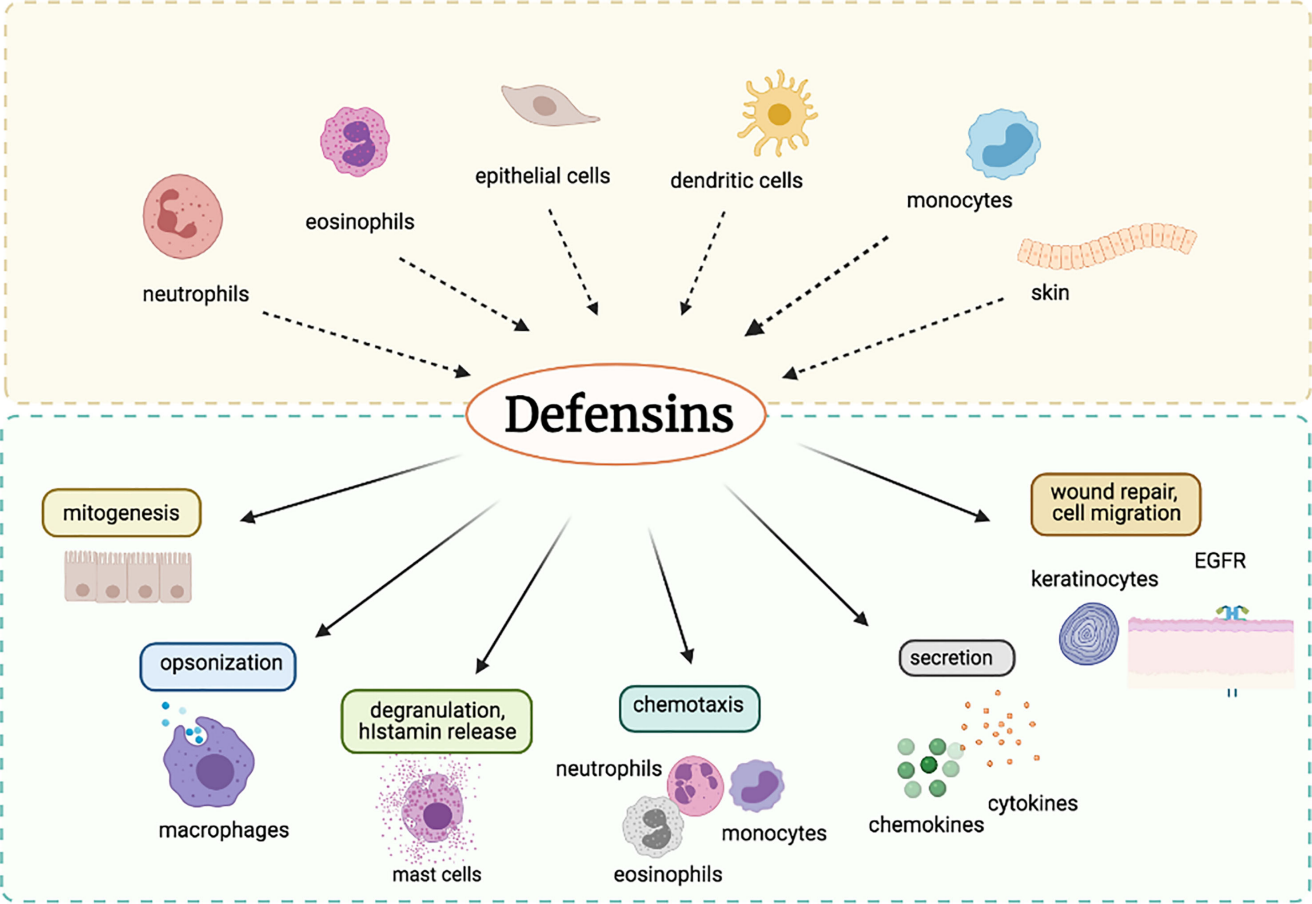
A schematic diagram for the role of defensins in the host immune system.

Defensins are a group of antimicrobial peptides (AMPs) that are an essential part of the innate immune system. Because of their broad-spectrum antimicrobial efficacy, they are imperative effector components in the defense of a host against infections (1–3). Based on configuration, defensins are categorized into two categories: α-defensins (α-helices) and β-defensins (β-sheets). Defensins are minute, cationic peptides that enable phagocytes, the skin, and the mucosa to fight bacteria. They also have a broad range of antimicrobial activity against viruses, mycoplasma, tumor, and fungi. They do have an amphipathic nature and acts on the membrane or envelopes the wall using their nature (4–6). The critical cellular secretors of these peptides include neutrophils and epithelial cells, but defensins are also generated by monocytes, macrophages, dendritic cells, and lymphocytes (7). According to previous studies, defensins are commonly dispersed among different body compartments in nearly all living organisms; however, they seem to be elevated in specific pathogenic body cells (8). These host defense peptides aid in the fight against bacterial, viral, and fungal infections *via* cells that produce them (7). Defensin peptides mostly destroy the structure of bacterial cell membranes as part of their action mechanism during which they inflict membrane permeabilization, which thereby results in the release of nutrients from the bacterial cell (9). They achieve this by binding to the membrane and forming destructive pores on the cell membrane. Defensins are induced by various stimuli (10). They are majorly synthesized and released from dendritic cells, monocytes, neutrophils, eosinophils, and epithelia cells. In addition to their antimicrobial activity, defensins are also actively involved in a range of immune-modulatory functions such as mitogenesis, cytokine release, and histamine release, as depicted in **Figure 1**.

- In the era of drug resistance, many emerging strains of pathogens (i.e., bacteria, fungi, parasites) are being found to be resistant to existing drugs, particularly against antibiotics (11, 12). This includes multidrug-resistant strains that are resistant to most of the existing drugs (13–15). In order to manage treatment of drug-resistant strains of pathogens, researchers are exploring alternatives to antibiotics (16, 17). One of the potential alternatives to antibiotics is protein-/peptide-based therapeutics. In the last two decades, there is a significant rise in the number of peptide-based therapeutics approved by the FDA (18–21). Some of the FDA-approved AMPs include poly(2-oxazoline)s, which are used as synthetic mimics of host defense peptides (22), as well as daptomycin, gramicidin, and colistin (23).

AMPs are one of the major classes of therapeutic peptides that are commonly used to kill microbial pathogens including the drug-resistant strain of pathogens (24, 25). In the past, numerous computational resources and methods have been developed for predicting AMPs including chemically modified AMPs (26–34). In addition to AMPs, a number of methods have been developed to predict peptides for killing a specific class of microorganism which include prediction of antibacterial, antituberculosis, antiviral, antifungal, and antiparasite peptides (35–41). Though these antimicrobial peptides are an alternative to small-molecule-based drugs, their toxicity, half-life, and allergenicity are major challenges (42–44). Thus, there is a need to explore a new class of AMPs called defensins, which are used by hosts to defend themselves from pathogens. These defensins have numerous advantages over AMPs as they are damage-associated molecular patterns (DAMPs) and released in the host itself. Due to this, they are less toxic and are highly tolerated by the body. They occur naturally and are recognized by pattern recognition receptors (PRRs) (45, 46). In the past, a number of methods have been developed for predicting defensins and their classes (47–50). We discussed the available tools in the section *Comparison With Existing Tools*.

In this paper, we describe a reliable method developed for predicting defensins with high precision. We systematically collected defensins, AMPs, and non-defensins from various sources to create the largest possible datasets. In this study, we tried to understand the differences and similarities between defensins and AMPs. We observed significant differences in defensins and AMPs. Thus, we developed models for discriminating antimicrobial peptides and defensins. In addition, we developed models for discriminating defensins and non-defensins. In order to help the scientific community, we developed a stand-alone software as well as a web server.

## 2 Materials and Methods

### 2.1 Creation of Datasets

Defensins were obtained from various sources that include previous studies (48–50), DRAMP2.0 (51), and CAMPR3 (30). We only collected experimentally validated defensin sequences which have antimicrobial activity. It was observed that defensins have a wide range of lengths (5–120 residues), but most of them (77.59% of the total sequences) have 10–60 residues. Thus, in this study, we removed all defensins which have number of residues less than 10 or more than 60 residues. We also removed sequences containing non-natural or non-standard amino acids (B, J, O, U, X, and Z). Finally, 1,036 unique defensins were obtained. These defensin sequences have been used to create two datasets, as described below.

#### 2.1.1 Defensins/AMPs or the Main Dataset

Our main dataset contains defensins as positive sequences and AMPs as negative sequences. As described above, we collected 1,036 defensins from different sources. We obtained 2,297 experimentally validated AMPs from the CAMPR3 database. Basically, we have taken all peptides excluding peptides of the defensin family. Similar to defensins, the sequence lengths were restricted between 10 and 60 residues. We also discarded sequences containing amino acids other than natural amino acids. In summary, our main dataset contains 1,036 experimentally validated defensins and 1,035 AMPs (or non-defensins).

#### 2.1.2 Defensins/Proteins or the Alternate Dataset

**Figure 2:**
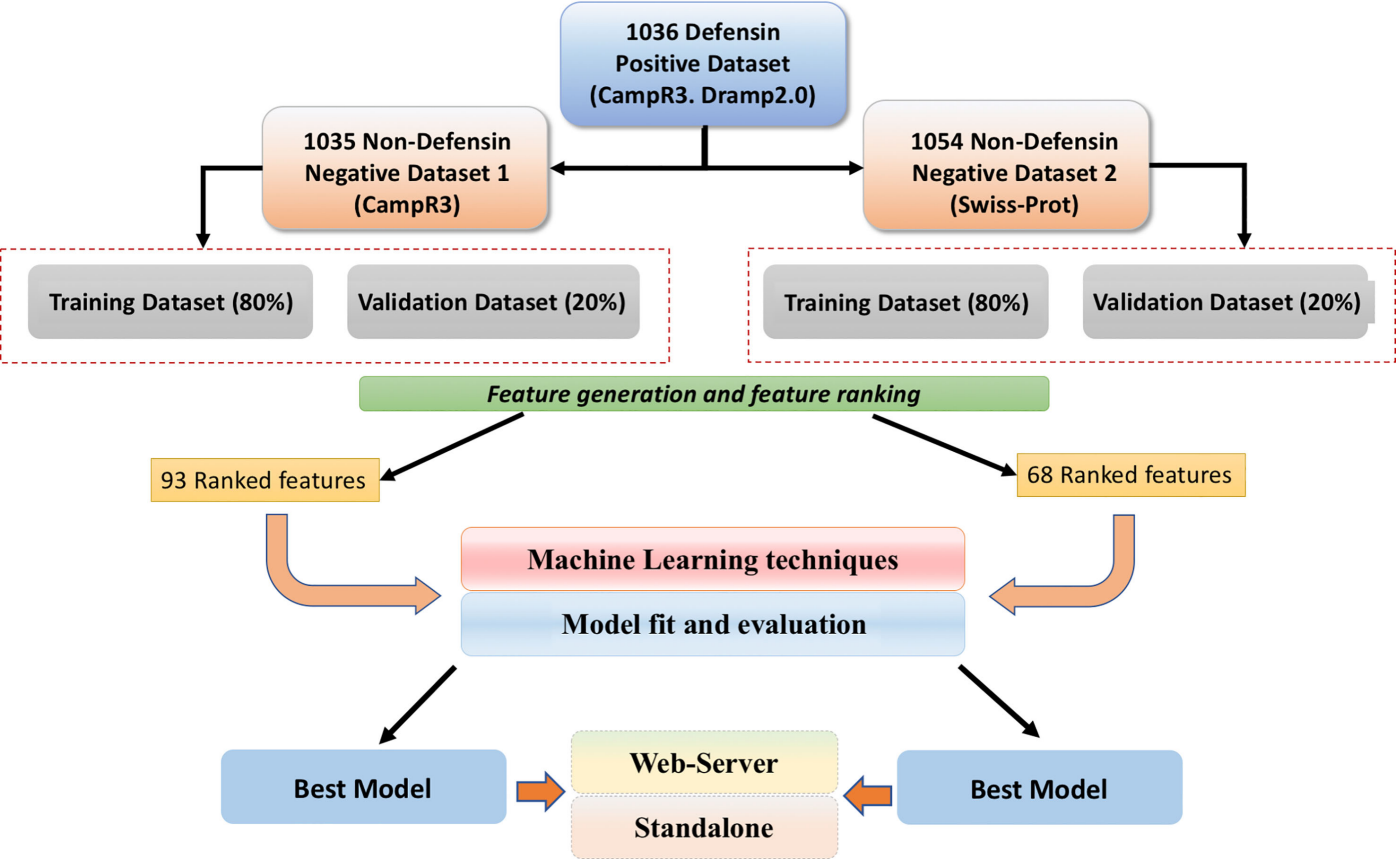
A brief workflow of the study.

Our alternate dataset has defensins and non-defensins. In order to obtain non-defensins, we searched Swiss-Prot (52) with following queries: “Non-AMPs” and “Non-Defensin” and “Not antibacterial” and “Not antifungal” and “Not antiviral” and “Not antiparasitic” and “Not antimicrobial” proteins. Initially, we obtained ~42,357 protein sequences, out of which we randomly selected 1,055 unique sequences having a number of residues between 10 and 60. In simple words, our alternate dataset contains 1,036 defensins and 1,054 non-defensin sequences as shown in **Figure 2**.

### 2.2 Sequence-Based Features

The stand-alone version of Pfeature (53) was used to calculate a variety of features from protein sequences in this analysis. Thousands of features/descriptors of protein or peptide sequences can be calculated using Pfeature. We applied the composition-based function module of Pfeature and created a vector of 8,968 features. Apart from these, we have also tried different composition features individually from Pfeature on both datasets. The corresponding results are shown in **Supplementary Tables 1, 2**.

### 2.3 Feature Selection and Ranking

Identifying an essential collection of features from the vast dimension of features is one of the main challenges of the study. We used the SVC-L1-based feature selection strategy, which incorporates the support vector classifier (SVC) with linear kernel, penalized with L1 regularization. SVC-L1 was chosen because it uses many methods to pick the right features from a vast number of feature vectors and is incredibly quick in comparison to other methods (54). Its main goal is to reduce the objective function, which takes into account the loss function and regularization. To minimize dimensions, the SVC-L1 algorithm chooses non-zero coefficients and, afterwards, applies the L1 penalty to choose appropriate features. During the optimization process, the L1 regularization generates sparse models by removing a few of the features from the model by setting the coefficients to zero. The sparsity is regulated by the “C” parameter, which is dependent on the number of features selected; the smaller the “C” value, the fewer features are selected. For parameter “C,” we used the default value of 0.01 (55). Subsequently, the significance of these features in classifying proteins was then evaluated using the software “feature selector.” The program “feature selector” ranks the features depending on the amount of time a feature is used to split data across all trees, using a DT-based algorithm called the Light Gradient Boosting Machine (56).

### 2.4 Machine Learning

In this study, several machine learning algorithms have been used to develop models for classification using Python’s library scikit-learn (57). It includes extra tree (ET), random forest (RF), logistic regression (LR), support vector machine (SVM), k-nearest neighbors (KNNs), and multilayer perceptron (MLP). Different hyperparameters corresponding to these classifiers were tuned using “GridSearch” and only the best results were incorporated.

### 2.5 Cross-Validation Techniques

In order to provide internal and external validation, we divide our datasets into training and validation sets in 80% and 20% ration, respectively. In case of internal validation, we used a five-fold cross-validation technique, where sequences in the training sets are first arbitrarily divided into five equivalent folds (58, 59). Thereafter, four of these folds are used for training and the remaining fold is used for testing. The procedure is replicated five times until each of the five folds has been used for testing at least once. Finally, the performance of the model is calculated by averaging the performance on the five folds. This is called internal validation where parameters are optimized on 80% training dataset to achieve the best performance. In order to validate the performance of our models, we evaluate the performance on 20% validation dataset, called external validation.

### 2.6 Evaluation Parameters

We used well-established evaluation criteria to assess the efficacy of various machine learning classification models. We used both threshold-dependent and independent parameters in this analysis like sensitivity (Sens), specificity (Spec), and accuracy (Acc). To assess the results of the models, a receiver operating characteristic (ROC) curve was plotted between sensitivity and 1 − specificity. Thereafter, we used the typical threshold-independent parameter AUROC (area under the ROC curve) values for assessment. The following equations were used to quantify these parameters:


$$Sens=\frac{TP}{P}\times 100$$

$$Spec=\frac{TN}{N}\times 100$$

$$Acc=\frac{TP+TN}{P+N}\times 100$$

$$MCC=\frac{TP\times TN-FP\times FN}{\sqrt{\left(TP+FP)(TP+FN)(TN+FP)(TN+FN\right)}}$$

where TP = true positive, FP = false positive, TN = true negative, and FN = false negative.

### 2.7 Architecture of the Web Server

To predict defensins and AMPs and defensins and non-defensins, a web server called “DefPred” (https://webs.iiitd.edu.in/raghava/defpred) was developed. HTML5, Java, CSS3, and PHP scripts were used to build the front end of the web server. It was built on responsive templates, which modify the size of the screen depending on the device. It works for virtually all electronic devices, including smartphones, tablets, and desktop computers.

## 3 Results

We conducted some preliminary analyses on the main and alternate dataset sequences to understand the preference of certain types of residues. Thereafter, the models were developed on the “main” and “alternate” datasets. A comprehensive detail about these analyses as well as the performance of the models is shown in the following sections.

### 3.1 Compositional Analysis

**Figure 3:**
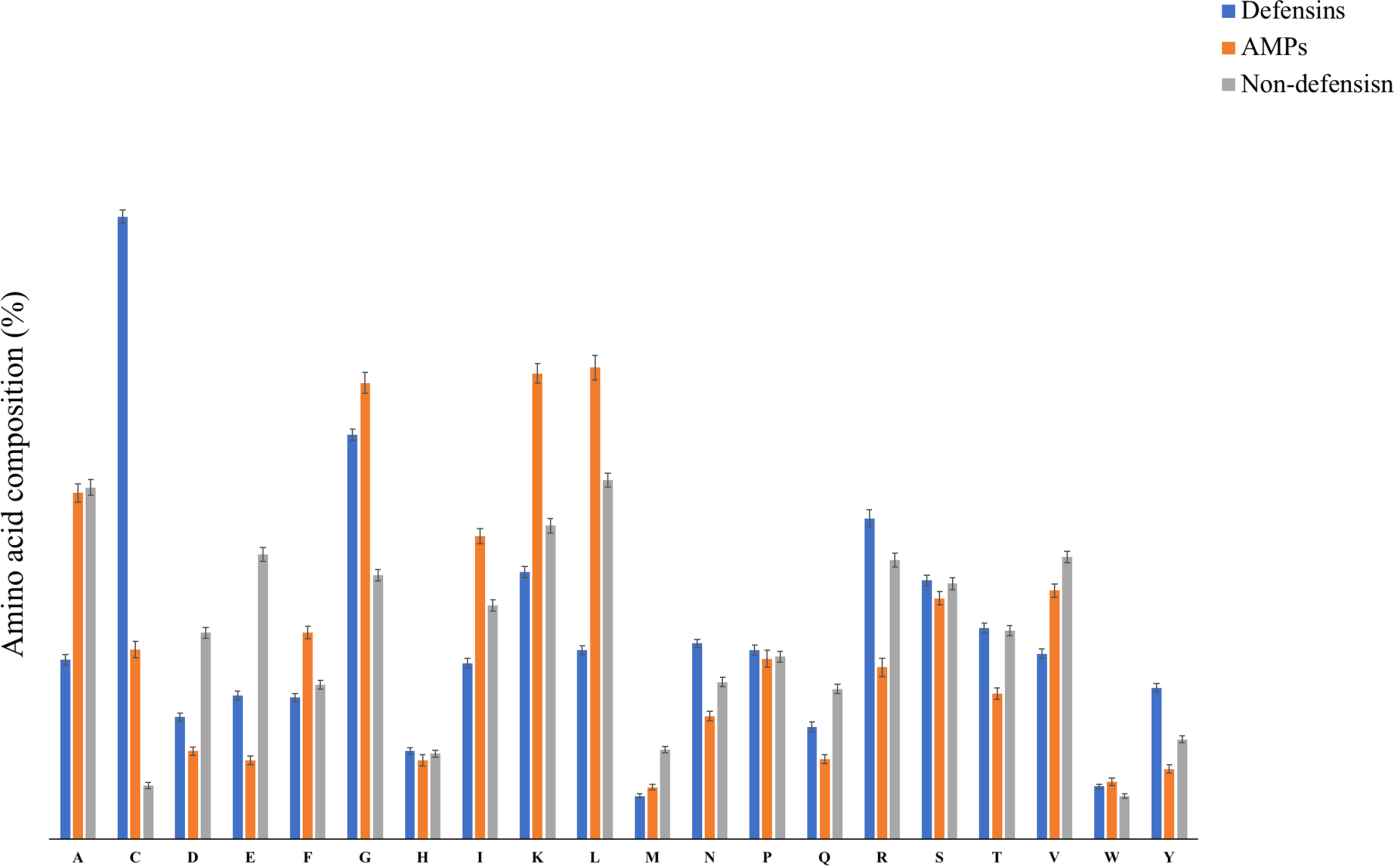
The average amino acid compositional analysis among defensins, AMPs, and non-defensins.

The amino acid composition (AAC) for defensins, AMPs, and non-defensin peptides was calculated. **Figure 3** depicts the typical amino acid composition of defensin, antimicrobial, and non-defensin peptides. As shown in **Figure 3**, defensins have a higher amino acid composition for certain types of residues (i.e., C, D, E, N, R, T, Y) in comparison to AMPs. In comparison to non-defensins, defensins have a higher amino acid composition for the following types of residues: C, G, R, and Y. Similarly, AMPs have a higher composition for certain types of residues (e.g., C, I, K, L) in comparison to non-defensins. These observations indicate that defensin and AMPs are different in terms of preference of residues, despite that both of them have antimicrobial activity. These observations indicate that antimicrobial peptide prediction is not suitable for predicting defensins as both have preference to different types of residues. Besides, we also conducted the “Mann–Whitney test” to determine the statistical significance among these three groups. We found that among 60 pairs, 54 were statistically significant (**Supplementary Table 3**). AMPs and non-defensins have non-significant amino acid residue pairs like A and W. AMPs and defensins have M residue as a non-significant pair. At the same time, non-defensins with defensins have F, H, and T as non-significant pairs.

### 3.2 Preferential Position Analysis

**Figure 4:**
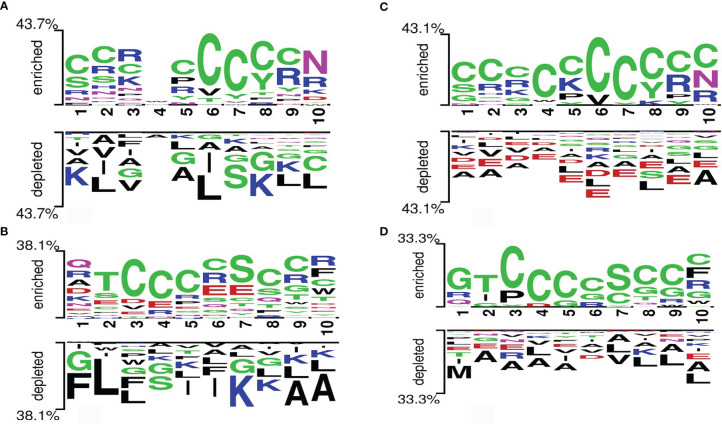
Two-sample logos generated from the **(A)** C-terminus (last 10 residues) of the main dataset, **(B)** N-terminus (first 10 residues) of the main dataset, **(C)** C-terminus (last 10 residues) of the alternate dataset, and **(D)** N-terminus (first 10 residues) of the alternate dataset.

In this analysis, the preference of a particular amino acid at a specific position in the protein string was studied. A two-sample logo (TSL) for the main and alternate datasets is represented in **Figure 4**. The most significant amino acid residue represents the relative abundance in the sequence. It is important to note that the first 10 positions represent the N-terminal residues of peptides, and the last 10 positions represent the C-terminus of peptides. We observed that the amino acid “C” was enriched at positions 1, 2, 3, 5, 6, 7, 8, and 9 of the C-terminus and at positions 3, 4, 5, 6, 8, and 9 of the N-terminus. Also, the amino acid “N” was enriched at position 10 of the C-terminus and “S” was enriched at position 7 of the N-terminus. However, the non-defensins show an abundance of “K,” “L,” and “A” at various positions in both C- and N-termini.

### 3.3 Development of the Prediction Models

#### 3.3.1 Feature Selection

Firstly, we computed a wide range of features using the Pfeature software. As all features are not important, so we removed all irrelevant features. Based on the SVC-L1 feature selection technique outlined in the *Materials and Methods* section, 93 important features for the main dataset and 68 important features for the alternate dataset (**Supplementary Tables 4, 5**) were identified from the 8,498 features. With the support of the “feature selector” tool, for each of these datasets, all features were ranked according to their normalized and cumulative scores.

#### 3.3.2 Machine Learning-Based Models on Selected Features

As outlined earlier, a total of 8,948 features fetched from Pfeature’s composition-based module were reduced to 93 (main dataset) and 68 (alternate dataset) features after applying the SVC-L1-based selection procedure. A range of machine learning classifiers including SVM, LR, KNN, RF, MLP, and ET were implemented on both these datasets. The performance of these models is illustrated in **Table 1**. Clearly, for the main dataset, SVM performs the best with AUROC and Matthews correlation coefficient (MCC) values of 0.98 and 0.88, respectively, at the training dataset. For the corresponding validation dataset, an AUROC of 0.97 and an MCC of 0.87 were obtained. LR was the second best model with 0.97 AUROC and 0.84 MCC at the training dataset and 0.97 AUROC and 0.85 MCC at the validation dataset. Similarly, for the alternate dataset, SVM was the best model with 0.99 AUROC and 0.94 MCC at the training dataset and 0.99 AUROC and 0.96 MCC at the validation dataset.

**Table 1:** The performance of the machine learning models on SVC-L1 selected features for both datasets.

Model	Hyperparameters	Training set					Validation set				
		Sens	Spec	ACC	AUROC	MCC	Sens	Spec	ACC	AUROC	MCC
Main dataset											
SVM	C = 2, g = 1, k = rbf	93.24	94.81	94.03	0.98	0.88	93.72	93.24	93.48	0.97	0.87
LR	C = 1	92.4	91.67	92.03	0.97	0.84	92.75	91.79	92.27	0.97	0.85
ET	ne = 30	93.73	94.08	93.9	0.98	0.88	93.24	93.72	93.48	0.97	0.87
RF	ne = 90	91.07	95.41	93.24	0.98	0.87	91.3	95.17	93.24	0.98	0.87
KNN	al = ball-tree, nn = 10, w = distance	92.52	94.32	93.42	0.97	0.87	92.27	90.82	91.55	0.96	0.83
MLP	a = identity, HL = 3, m = 100, s = adam	92.4	89.73	91.07	0.95	0.82	93.72	87.92	90.82	0.96	0.82
Alternate dataset											
SVM	C = 2, g = 0.5, k = rbf	95.05	98.46	96.77	0.99	0.94	97.1	99.05	98.09	0.99	0.96
LR	C = 10	94.93	97.86	96.41	0.99	0.93	94.69	98.58	96.65	0.99	0.93
ET	ne = 50	94.09	98.93	96.53	0.99	0.93	94.69	99.53	97.13	0.99	0.94
KNN	al = brute, nn = 10, w = distance	92.88	98.22	95.57	0.99	0.91	94.69	98.58	96.65	0.98	0.93
RF	ne = 70	95.66	97.27	96.47	0.99	0.93	96.14	97.16	96.65	0.99	0.93
MLP	a = tanh, HL = 10, m = 100, s = adam	92.4	97.86	95.16	0.98	0.9	93.72	98.1	95.93	0.98	0.92

g, gamma; ne, n_estimators; k, kernel; a, activation; HL, hidden layer size; s, solver; al, algorithm; w, weight; m, max_iter; nn, n_neighbors.

#### 3.3.3 Machine Learning-Based Models on Top-Ranked Selected Features

**Figure 5:**
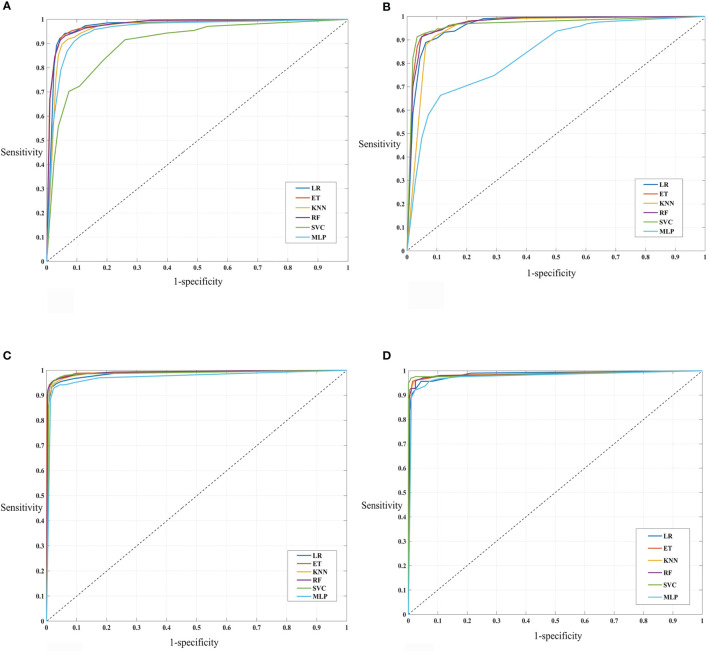
AUROC plots **(A)** main (top 60 selected features on the training datasets), **(B)** main (top 60 selected features on the validation datasets), **(C)** alternate (top 50 selected features on the training datasets), and **(D)** alternate (top 50 selected features on the validation datasets).

In addition to the development of prediction models over complete selected features, we assessed the significance of various feature sets. The goal was to determine the feature set with minimal features that can reliably distinguish defensins with AMPs and non-defensins with high AUROC and accuracy. As a result, we created various models based on the top (10, 20, 30,…, 93) features in the case of the main dataset and top (10, 20, 30, …, 68) features in the case of the alternate dataset, respectively, and tested them on the training and validation datasets. The complete results corresponding to these are provided in **Supplementary Tables 3**, **4** highlights the performance of the various models. As seen from the results, the best features were identified, i.e., the top 60 for the main and the top 50 for the alternate dataset. SVM (training: 0.98 AUROC, 0.88 MCC and validation: 0.98 AUROC, 0.88 MCC) is the best model for the main dataset followed by LR (training: 0.96 AUROC, 0.82 MCC and validation: 0.97 AUROC, 0.83 MCC). Similarly, for the alternate dataset, SVM (training: 0.99 AUROC, 0.93 MCC and validation: 0.99 AUROC, 0.96 MCC) is the best model followed by LR (training: 0.99 AUROC, 0.91 MCC and validation: 0.98 AUROC, 0.90 MCC) as shown in **Table 2** and **Figure 5**.

**Table 2:** The performance of machine learning models on top 60 features for main dataset and top 50 features for alternate dataset.

Model	Hyperparameters	Training dataset					Validation dataset				
		Sens	Spec	ACC	AUROC	MCC	Sens	Spec	ACC	AUROC	MCC
Main top 60											
SVM	C = 2, g = 1, k = rbf	89.26	96.74	93	0.98	0.86	90.82	97.1	93.96	0.98	0.88
LR	C = 0.1	86.85	93.24	90.04	0.96	0.8	88.89	93.72	91.3	0.97	0.83
ET	ne = 50	92.4	95.41	93.9	0.98	0.88	92.4	95.41	93.9	0.98	0.88
RF	ne = 60	91.68	95.29	93.48	0.98	0.87	91.3	94.69	93	0.98	0.86
MLP	a = tanh, HL = 17,m = 100, s = adam	74.79	70.77	72.78	0.85	0.46	91.79	91.3	91.55	0.96	0.83
KNN	al = ball-tree, nn = 10, w = distance	91.8	93	92.4	0.97	0.85	91.79	90.34	91.06	0.96	0.82
Alternate top 50											
SVM	C = 2, g = 1, k = rbf	95.17	97.98	96.59	0.99	0.93	97.1	99.05	98.09	0.99	0.96
LR	C = 1	95.54	95.02	95.28	0.99	0.91	95.65	95.73	95.69	0.98	0.91
ET	ne = 40	95.17	98.22	96.71	0.99	0.93	95.65	98.58	97.13	0.99	0.94
KNN	al = ball-tree, nn = 9, w = distance	94.33	97.86	96.11	0.99	0.92	95.65	98.1	96.89	0.98	0.94
RF	ne = 50	95.3	98.22	96.77	0.99	0.94	96.65	97.63	96.65	0.99	0.93
MLP	a = tanh, HL = 15, m = 100, s = adam	92.64	97.75	95.22	0.98	0.91	92.27	97.63	94.98	0.98	0.9

g, gamma; ne, n_estimators; k, kernel; a, activation; HL, hidden layer size; s, solver; al, algorithm; w, weight; m, max_iter; nn, n_neighbors.

### 3.4 Comparison With Existing Methods

We have also compared our models developed in this study with the methods developed in the past. As shown in **Table 3**, these methods have been developed over the years on different datasets where size and type are different. Thus, it is not possible to compare these methods directly with other methods. In previous studies, defensin peptides were obtained either from the Defensins Knowledgebase, developed in 2006 (61), or from Swiss-Prot (62). One of the limitations of previous studies is the size of the dataset. In this study, we have taken the largest possible dataset to develop reliable models where data were obtained from different sources. In addition, we created two datasets called the main and alternate datasets to discriminate defensin from antimicrobial peptide and non-defensins. Our web service not only allows to predict defensin but also to scan defensin peptides in proteins as well as to design highly efficient defensins. In contrast, most of the web services developed in the past are inactive. This justifies the development of this new method which will complement existing methods.

**Table 3:** Describing the major components of the existing methods and DefPred such as the source of the dataset, size of data, major features, type, and performance.

Study	Source of dataset	Size of data	Major features	Classifier used	Type	Accuracy	Web server availability, status	PMID
49	Defensin Knowledgebase	286 P	ID_RAAA	Jackknife test	Prediction	91.36%	No	19591890
60	PubMed, iHOP, UniProt, HubMed	238 P, 238 N	RQA descriptors	RF	Classification	78.12%	No	Not-available
47	NCBI, UniProt	383 P, 383 N	AAC, DPC, PSAAC	SVM	Classification	99%	Yes, inactive	22670676
48	Defensin Knowledgebase	333 P	iDEF-PseRAAAC	SVM	Prediction	85.59%	Yes, inactive	26713618
50	Defensin Knowledgebase	328 P	iDEF-PseRAAC	SVM	Prediction	91.16%	Yes, active	31391777
DefPred	CAMPR3, DRAMP2.0, Defensin Knowledgebase, Swiss-Prot	1,036 P, 1,035 N (main); 1,036 P, 1,054 N (alternate)	Selected features	SVM	Prediction	93.96% (main), 98.09% (alternate)	Yes, active	Not-available

P, positive sequence; N, negative sequence.

### 3.5 The Web Server “DefPred”

We built a user-friendly prediction web server that incorporates various modules to predict defensin proteins in order to support the scientific community. The prediction models of the study are applied in the web server. Based on the score of the prediction models at a different threshold, users will predict whether a query peptide is defensin or non-defensin. Predict, Protein-scan, Design, Downloads, and Algorithm are the five major modules in the web server. The user can distinguish defensins from non-defensin peptides using the “Predict” module. The positive and negative datasets used in this analysis are both available for download in FASTA format. HTML, Java, and PHP scripts were used to build the web server “DefPred.” A detailed description of these modules is provided below. The Predict module predicts whether the submitted protein sequence is defensin or not. Users can submit multiple peptides in FASTA format in the box or can upload the file containing the same. This module allows the user to predict using model-1 developed on the main dataset to predict defensins from AMPs. Model-2 was developed to predict defensins and non-defensins. The Design module allows the user to generate all possible analogs for a sequence and then rank these peptide sequences based on their scores. This allows the user to identify the best analog of defensin. The Scan module is developed to identify regions in a protein that have defensin-like properties. In order to serve the community, we have developed the stand-alone software in Python. We have also provided a stand-alone facility in the form of Docker technology. This stand-alone software is integrated into our package “GPSRdocker,” which can be downloaded from the site https://webs.iiitd.edu.in/gpsrdocker/ (63).

## 4 Discussion

Antibiotic resistance is emerging among microbes throughout the world, and current treatments are ineffective to treat drug-resistant microorganisms. The fear of a post-antibiotic age, with rising pathogen drug resistance, necessitates the development of alternatives to traditional antibiotics or small molecule-based treatments. AMPs are a class of potential agents with curative prospects due to their diverse therapeutic properties. The innate immune systems of several organisms rely heavily on these evolutionarily conserved molecules. Defensins are a special class of AMPs that have a wide range of functions and use several modes of action, making them less likely to be drug resistant (8, 64). Moreover, the differences in the mechanism of microbicidal action of defensins from other antibiotics make them beneficial in fighting infections when used in tandem with conventional antibiotic treatments (65). Naturally existing defensins are efficient, non-toxic microbicides that might be effective for treating infections caused by antibiotic-resistant pathogens. Recent studies have suggested that they achieve this by damaging bacterial cell membranes but not mammalian cell membranes. With this information, developing next-generation defensins with enhanced biological activity profiles is a plausible objective that will allow defensins to be employed to augment human health in the near future. New antimicrobials with defensin-based bactericidal and immunomodulatory characteristics may be effective in conjunction with conventional antibiotic therapy against drug-resistant bacteria while also increasing survival from common infections (65). Furthermore, previous research has demonstrated that defensin and antibiotic combinations may be utilized synergistically to battle infections, including biofilms, permitting for lower dosages of both drugs while still improving treatment efficacy (66–69). The advancement in *in-silico* research particularly in the field of bioinformatics has led to the identification and delineation of properties of defensins that enable them to exert their diverse range of biological activities. However, since defensins and AMPs have highly similar nature, it is difficult to distinguish defensins and thereby challenging to develop solely defensin-based therapeutics.

Our study addresses this issue by proposing state-of-the-art machine learning models which can be employed to discriminate and predict defensins from other AMPs and defensins from other proteins (non-defensins). Additionally, since the dataset is crucial in machine learning as well as for a robust *in-silico* prediction model, we created a very detailed and up-to-date dataset using updated repositories. To better understand the structure and positional preference of defensins, TSL and compositional analytical experiments were conducted. In previous studies, defensins have been found to be high in cysteine (C) amino acid (7) which is consistent with our findings. The properties of the experimentally validated defensins present in the literature were utilized for developing various prediction models. The program “Pfeature” was used to generate 8,968 features from sequence data. The SVC-L1 of the scikit package was used to pick selected features, which were then ranked using feature selector methods. The compositional analysis demonstrated that some types of residues such as C, R, N, L, and Y are preferred in defensins, whereas others such as M are not. This was also corroborated from one of the top-ranked selected features AAC_C which denotes the amino acid composition of cysteine in a protein sequence. AAC_C ranked first in the main and second in the alternate dataset. Some other high-ranked features included CeTD_SA1 which is composition-enhanced transition and distribution of group 1 (A, L, F, C, G, I, V, W) for solvent accessibility attribute, and PAAC1_E is the pseudo-amino acid composition of glutamic acid in the main dataset (**Supplementary Table 4** and **Table 2**). In the case of the alternate dataset, a few top-ranked features were CeTD_SS1, which is a composition of group 1 (A, L, F, C, G, I, V, W) residue for the secondary structure attribute, and BTC_T, which is the total bond composition present in the sequence (**Supplementary Table 5** and **Table 2**). Amino acid composition of cysteine is common in both main and alternate datasets, indicating that defensins outstand with more “C” content (**Figure 3**). It is worth noting that new feature selection strategies picked 93 features for the main and 68 features for the alternate dataset, which include the abovementioned features. In our work, we used these 93 and 68 features to build the two classification models. Furthermore, a five-fold cross-validation technique was used to validate the performance of different models based on the top-ranked features. We wanted a minimal set of features with the least amount of performance loss to prevent over-optimization of the models. For the final classification models, for the main and alternate datasets, we chose the top 60 and top 50 features, respectively. Model-1, which utilized the main dataset, is a SVM classifier that achieved maximum performance of 0.98 AUROC and 0.88 MCC in the training dataset and 0.98 AUROC and 0.88 MCC in the validation dataset for classifying defensins from AMPs, whereas model-2, which used the alternate dataset, classified defensins from non-defensins. Model-2 is also a SVM classifier which performed best on the training and validation datasets with AUROC of 0.99 and MCC of 0.93 and AUROC of 0.99 and MCC of 0.96, respectively.

Despite numerous improvements, there are a few limitations of this study. The current study aimed to develop a prediction method for identifying defensins/AMPs and defensins/non-defensins. To achieve this, we used the sequence data from all available species such as mammals, plants, and insects due to the small number of experimentally validated defensins, although the ideal process to develop a host-specific method for predicting defensins should contain data from the concerned host only. Additionally, our models do not account for structural properties such as secondary structure details, surface accessibility rating, and disulfide bond information. Furthermore, for prediction, our models ignore information regarding post-translational modifications (e.g., terminus modification, incorporation of chemical moieties, glycosylation, and phosphorylation). Although a systematic effort has been made in this analysis to create the best possible models under the current conditions, it is expected that the future research will be able resolve these issues in order to improve prediction.

Finally, in order to serve the scientific community, we have developed a web server named “DefPred” as well as the stand-alone version which incorporated our best models. The stand-alone version is Python-based and offers numerous options to the user. On the other hand, the associated server is user-friendly and compatible with multiple screens such as laptops, android mobile phones, iPhone, and iPad. We have also provided a stand-alone facility in the form of Docker technology. This stand-alone software is integrated into our package “GPSRdocker,” which can be downloaded from the site https://webs.iiitd.edu.in/gpsrdocker/ (63). We anticipate that this work will benefit researchers working in the area of vaccine designing and also enable a deeper understanding of immune defense response.

## 5 Conclusion

In this work, we have presented a prediction server “DefPred” for the identification and classification of defensins. It possesses two models “model-1” and “model-2” for the classification of defensins from other AMPs (the main dataset) and defensins from any random proteins (the alternate dataset), respectively. Both models have been created from different datasets that are available on the web server. The web server employs SVM supervisory models in both datasets. Around 9,000 features have been taken into account, and after feature selection and ranking, 98 features for the main dataset and 68 features for the alternate dataset have been selected. Furthermore, among them, the best models for the main and alternate datasets were obtained at the top 60 and top 50, respectively. The present work is an attempt to provide a platform for addressing this important aspect of defensin prediction. To facilitate the scientific community in developing better methods for the prediction of defensins, we have provided our datasets used in the present study. Also, we have provided the stand-alone version for “DefPred.”

## Data Availability Statement

The dataset is available at: https://webs.iiitd.edu.in/raghava/defpred/dataset.php.

## Author Contributions

Concept and design of the study: DK and GR. Acquisition of the data: DK. Implementation of the algorithm: DK, RS, and GL. Analysis and interpretation of the data: DK, SP, CA, and GR. Drafting of the article: DK, CA, and GR. Web server interface: SP and DK. Final approval of the version to be submitted: DK, SP, CA, RS, GL, and GR.

## Funding

The authors are thankful to IIITD and DBT for fellowships and financial support.

## Conflict of Interest

The authors declare that the research was conducted in the absence of any commercial or financial relationships that could be construed as a potential conflict of interest.

## Publisher’s Note

All claims expressed in this article are solely those of the authors and do not necessarily represent those of their affiliated organizations, or those of the publisher, the editors and the reviewers. Any product that may be evaluated in this article, or claim that may be made by its manufacturer, is not guaranteed or endorsed by the publisher.
